# Effect of bioelectrical impedance analysis-guided dry weight adjustment, in comparison to standard clinical-guided, on the sleep quality of chronic haemodialysis patients (BEDTIME study): a randomised controlled trial

**DOI:** 10.1186/s12882-019-1405-z

**Published:** 2019-09-02

**Authors:** Sethanant Sethakarun, Sutachard Bijaphala, Chagriya Kitiyakara, Sarinya Boongird, Pariya Phanachet, Sirimon Reutrakul, Kwanchai Pirojsakul, Arkom Nongnuch

**Affiliations:** 1 Division of Nephrology, Department of Medicine, Ramathibodi Hospital, Mahidol University, 270 Rama VI Road, Thung Phaya Thai, Ratchatewi, Bangkok, 10400 Thailand; 2 Division of Nephrology, Department of Medicine, Vichaiyut Hospital, 53 Set Siri Road, Sam Sen Nai, Phaya Thai, Bangkok, 10400 Thailand; 3 Division of Nutrition and Biochemical Medicine, Department of Medicine, Ramathibodi Hospital, Mahidol University, 270 Rama VI Road, Thung Phaya Thai, Ratchatewi, Bangkok, 10400 Thailand; 4 Division of Endocrinology and Metabolism, Department of Medicine, Ramathibodi Hospital, Mahidol University, 270 Rama VI Road, Thung Phaya Thai, Ratchatewi, Bangkok, 10400 Thailand; 5 Division of Endocrinology, Diabetes and Metabolism, Department of Medicine, University of Illinois at Chicago, Chicago, Illinois USA; 6 Division of Nephrology, Department of Paediatrics, Faculty of Medicine Ramathibodi Hospital, Mahidol University, Bangkok, Thailand

**Keywords:** Haemodialysis, Sleep quality, Actigraphy, Bioelectrical impedance analysis (BIA), Dry weight, Adjustment

## Abstract

**Background:**

Sleep disturbance is common among chronic haemodialysis patients, which leads to poor quality of life, in addition to increased instances of morbidity and mortality. Hypervolemia has been linked to sleep problems observed in chronic haemodialysis patients, which suggests that optimising one’s fluid status could improve the sleep quality of this patient group. In our study, we subjectively examined and objectively measured sleep parameters, using actigraphy recordings, the Pittsburgh Sleep Quality Index (PSQI) questionnaire, and Epworth Sleepiness Scale (ESS), in order to compare bioelectrical impedance analysis (BIA)-guided and standard clinical-guided dry weight adjustment.

**Methods:**

We randomly selected 19 chronic haemodialysis patients with subclinical hypervolemia, defined as a clinically euvolemic status, despite the ratio of extracellular water to total body water being more than 0.4 in BIA. Furthermore, these patients, who were poor sleepers (PSQI > 5), were assigned to either a BIA-guided dry weight group (BIA group) or a standard clinical-guided one (clinical group). The primary outcome was changes in sleep actigraphy parameters between the groups at 1, 3, and 6 months. Changes observed in the PSQI and ESS score between the two groups over the same period of time were the secondary endpoints.

**Results:**

The mean age of the participants was 63.53 ± 11.12 years, and 42% of them were male. All sleep parameters measured by means of actigraphy were not significantly different between the two groups. Interestingly, at 3 and 6 months, the subjective sleep quality significantly improved in the BIA group, as reflected by a greater decline in the PSQI score, in comparison with the clinical group (3 months: mean difference − 1.82 [− 3.13 to − 0.51], *P* = 0.006; 6 months: mean difference − 3.16 [− 4.49 to − 1.83], *P* <  0.001). However, sleepiness assessed by the ESS was not significantly different between the groups throughout the study.

**Conclusions:**

Optimisation of the fluid status by employing BIA did not improves sleep actigraphy parameter, however, it significantly ameliorates the subjective sleep quality of chronic haemodialysis patients. This observation should be further explored in larger samples and longer clinical trials.

**Trial registration:**

This trial was registered at ClinicalTrials.gov (NCT02825589) on July 7, 2016.

**Electronic supplementary material:**

The online version of this article (10.1186/s12882-019-1405-z) contains supplementary material, which is available to authorized users.

## Background

The majority of chronic haemodialysis (HD) patients suffer from one or more sleep disorders, which are often under-recognised and/or under-treated [1]. Insomnia and obstructive sleep apnoea (OSA) are the most common disorders in HD patients [2]. Moreover, the sleep quality of the said patients is significantly poorer compared to chronic kidney disease patients [3, 4]. Poor sleep quality is associated with impaired daytime functioning, impaired quality of life [5], and increased morbidity [6] and mortality [7] in patients with HD. In addition, hypervolemia is noted to be extremely common in such patients, which can contribute to the worsening of OSA owing to overnight fluid shift from the extremities to the chest and head [8]. A previously conducted study reported an improvement regarding sleep apnoea with the help of nocturnal HD [9], which places emphasis on the role played by excess fluid in case of sleep disorders. Furthermore, a recent study reported that an increase in predialysis volume status measured with bioelectrical impedance analysis (BIA) was associated with fewer hours of sleep [10], which leads to the concept of sleep quality improvement with better volume control in chronic HD patients.

Sleep quality assessment can be evaluated both subjectively and objectively. The Pittsburgh Sleep Quality Index (PSQI) is a widely used questionnaire used to assess patients’ quality and patterns of sleep, which comprises 7 domains, wherein each domain yields a score of 0–3. A PSQI total global score is obtained by summarising the 7 domains’ scores, and a total score of more than 5 points indicates poor sleep quality. Another questionnaire, the Epworth Sleepiness Scale (ESS), is utilised to assess daytime sleepiness. Polysomnography (PSG), which provides an objective assessment of sleep, including evaluation of the presence and severity of OSA, is considered to be the gold standard testing method but can only be performed in a sleep laboratory. In addition, PSG might not represent the habitual quality and patterns of sleep. Alternatively, objective sleep measurement can be performed using actigraphy, employing an accelerometer that is worn on the wrist for at least 3 days in a patient’s home environment. Actigraphy detects the movement of patients during sleep and while awake, reflecting their sleep quality and sleep duration. Actigraphy results are typically strongly correlated to PSG results [11, 12] and useful to evaluate habitual sleep pattern.

**Fig. 1:**
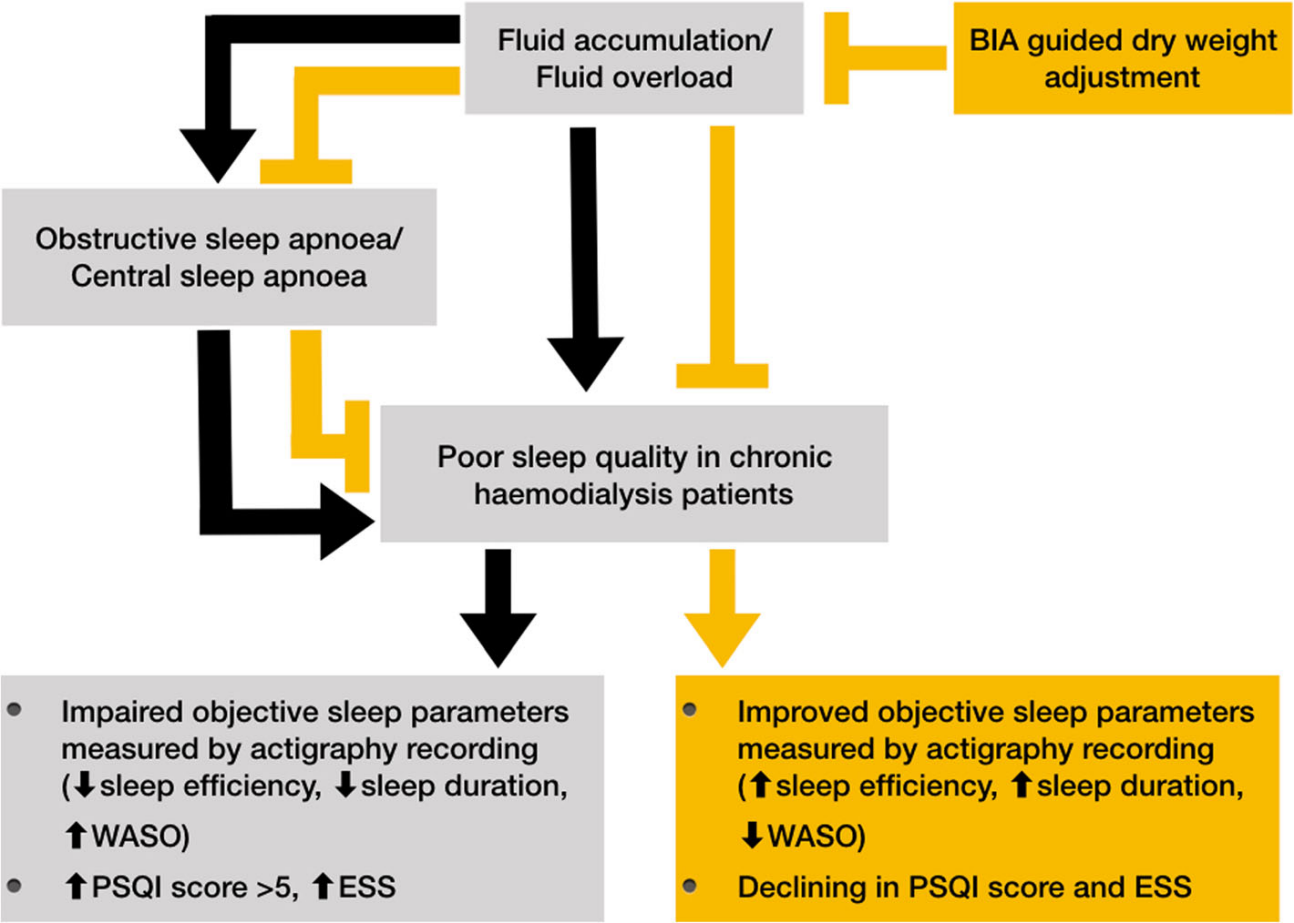
Hypothesised associations of fluid overload and poor sleep quality in chronic haemodialysis patients and the way BIA-guided dry weight adjustment could intervene this process in our study. Abbreviations: BIA, bioelectrical impedance analysis; HD, haemodialysis; WASO, wake after sleep onset; PSQI, Pittsburgh Sleep Quality Index; ESS, Epworth Sleepiness Scale

To expand the understanding of the influence of improved volume control on one’s sleep quality, we performed a randomised controlled trial and evaluated the effects of BIA-guided dry weight adjustment, compared to standard clinical-guided dry weight adjustment, on the objective and subjective assessment of sleep parameters, which were measured by means of actigraphy, the PSQI, and the ESS in chronic HD patients. We hypothesised that the adjustment of dry weight by employing BIA would improve the measurement of the subjective and objective sleep quality, in comparison to standard clinical evaluation of subclinical hypervolemic chronic HD patients. The hypothesised associations observed in our study have been depicted in Fig. 1.

## Methods

### Study design and samples

This was a multicentre, prospective, randomised (1:1), single blind (patients blind), comparator-controlled, parallel group study. This research was conducted at the HD unit of Ramathibodi Hospital, Somdech Phra Debaratana Medical Center, and at Vichaiyut Hospital between July 2016 and February 2017. The study protocol was in accordance with the Declaration of Helsinki and was approved by the ethics committee of each study site. Furthermore, this study was registered at ClinicalTrials.gov (NCT02825589) on July 7, 2016. The inclusion criteria required the participants to be 18 years old or older, with poor sleep quality (defined as a baseline PSQI score greater than 5) and subclinical hypervolemia, which was defined as a clinically euvolemic status on physical examination. However, the ratio between extracellular water and total body water (ECW/TBW) was more than 0.4, as measured through BIA, and the patients had to undergoing HD thrice weekly. Moreover, the patients, who were bedridden, suffered from clinically significant cognitive dysfunction, inadequate urea clearance (equilibrated Kt/V less than 1.2 per session) with unstable haemodynamics. However, psychiatric diseases were excluded in this regard. All the participants of this study provided written informed consent prior to their participation.

### Study protocol

For allocation of the participants, a computer-generated randomisation list was utilised. The allocation sequence of a computerised random number was concealed from the researcher responsible for enrolling and assessing participants and stored in sequentially numbered, sealed, opaque envelopes. Randomisation sequence generation and allocation concealment were conducted by a statistician who was unaware of the study protocol. Subsequently, participants were assigned randomly, using simple randomisation with a 1:1 allocation ratio, following which they were divided into either the BIA-guided dry weight adjustment group (BIA group) or the standard clinical-guided one (clinical group) and were blinded to the allocated arm as well. The BIA group underwent multi-frequency BIA just prior to a HD session at the baseline, 3, and 6 months for obtaining proper dry weight. An estimated amount of excess fluid measured in litres (L) was set as the target additional ultrafiltration (UF), and dry weight was decreased by 0.2 kg (kg) per week until the target dry weight was attained. If the patients developed symptoms indicating hypovolemia, such as intradialytic hypotension (IDH), cramps, or dizziness, we stopped reducing the dry weight and used the previous weight in which the patients showed no symptoms of hypovolemia as the target dry weight for these patients. In the clinical group, dry weight adjustment was performed on the basis of the clinical parameters, including blood pressure, physical examination, or chest X-ray. Both the groups underwent HD thrice weekly with an unchanged prescription, unless a clinical indication was observed. Some patients were administered benzodiazepines, which could affect their sleep. However, these medications were not changed during the study period. All the participants were assessed for their sleep parameters by employing actigraphy as well as the Thai versions of the PSQI and ESS at the baseline, 1 month, 3 months, and 6 months after randomisation.

### Data collection

Baseline data collection included conducting a brief interview in order to collect general demographic data and assess the patients’ body mass index (BMI), predialysis pulse rate, and blood pressure. A history of physician-diagnosed medical illnesses, causes of end-stage renal disease (ESRD), or current medication use were obtained from the medical record review. Dialysis treatment data including dialysis vintage (duration of dialysis), mode of dialysis, and dialysis shift were also derived from the dialysis records of the patients. Moreover, laboratory data were collected from the participants prior to a dialysis session at the time of baseline examination. The beta-2 microglobulin (B2M) level, a representative of middle molecule uremic toxin, was also acquired. In addition, we obtained the most recent equilibrated Kt/V (eKt/V) data from the dialysis records, which reflect HD adequacy. The minimum target of eKt/V was 1.2 for HD conducted thrice weekly.

### Bioelectrical impedance analysis (BIA) and excess fluid

We performed whole-body multi-frequency BIA using a device that transmits electrical currents at frequencies of 5, 50, 250, 500, and 1000 kHz (InBody720; Biospace, Seoul, Korea). Furthermore, BIA measurement was conducted just prior to the patients’ HD sessions. The patients wore light clothing without socks or shoes and stood on the electrodes in the BIA device, while holding two other electrodes in each hand in a tetrapolar configuration. We tested for a ratio of extracellular water to total body water (ECW/TBW) greater than 0.4, which would indicate that patients still had excess fluid in their bodies. In addition, we used a formula developed by Chamney and colleagues to estimate predialysis excess fluid in litres [13].

### Sleep assessment through Actigraphy

Participants wore an Actiwatch 2 activity monitor (Philips Respironics, Bend, Oregon) on their non-active vascular access wrist for 7 days. These monitors employ highly sensitive omnidirectional accelerometers to measure the number of wrist movements in 30 s-epochs. The software scores each 30 s-epoch as sleep or wake based on a threshold of activity counts estimated using activity within the epoch being scored, in addition to the epochs 2 min before and after that particular epoch. Furthermore, bedtime and wake time were set by the researcher using event markers, with sleep log data as well as an in-person review of the sleep timing with the participants when they returned the watch. Sleep duration was defined as the amount of actual sleep obtained at night, whereas sleep efficiency (a measure of sleep quality) was defined as the percentage of time in bed spent sleeping, and wake after sleep onset (WASO) was defined as the number of minutes of wakefulness occurring after defined sleep onset. These three parameters were calculated using Actiware 6.0 software, which was supplied by the manufacturer. For each participant, the mean across all available nights was considered. At least 6 days of actigraphy recording were available for 90% of the participants in this study, and the remaining 10% had 4–5 days of the same.

### Subjective sleep assessment

The PSQI is a questionnaire used to measure patients’ subjective sleep quality over the month preceding randomisation. It comprises 7 domains of questions: subjective sleep quality, sleep latency, sleep duration, habitual sleep efficiency, sleep disturbances, use of sleep medication, and daytime dysfunction. The answers are scored using a scale ranging from 0 to 3 points, with higher scores reflecting poorer sleep. A global score of more than 5 points indicates poor sleep quality. In addition, we measured daytime sleepiness using the ESS, wherein patients were asked to rate, on a 4-point scale (0–3), the probability of falling asleep in 8 daily situations. The total scores obtained ranged from 0 to 24, with higher scores reflecting more daytime sleepiness. We used the Thai version of the PSQI [14] and ESS [15], which had been validated. Moreover, standardised interviews were performed by a physician.

### Study endpoint

The primary endpoint of our study was the mean difference observed with respect to the changing of the objective sleep parameters measured through actigraphy (sleep efficiency, sleep duration, and WASO) between the two aforementioned groups at 1, 3, and 6 month(s). The secondary endpoints were the mean difference in the changes observed regarding the subjective sleep quality measured in the PSQI and ESS scores between the groups at 1, 3, 6 month(s).

### Sample size determination

We calculated the sample size on the basis of the data garnered from a previous cross-sectional study, which ascertained that the mean sleep efficiency measured through actigraphy in HD patients was 54.4%, whereas the clinically significant difference in sleep efficiency was 20% [16]. With a 10% dropout rate, the total sample size was 24 patients, with 80% power to detect the hypothesised difference between the two groups (two-sided α = 0.05). No previous longitudinal study has examined the way in which the interval of dry weight adjustment affects the sleep quality of chronic HD patients. We hypothesised that the said effect of dry weight adjustment could improve patients’ sleep quality in several months. In this study, the sample size was estimated as follows:

$$ r=\frac{n_2}{n_1},\Delta  ={\mu}_1-{\mu}_2,\sigma = SD,\kern0.5em {z}_{1-\frac{\alpha }{2}}=1.96,{z}_{1-\beta }=0.84 $$

$$ {n}_1=\frac{{\left({z}_{1-\frac{\alpha }{2}}+{z}_{1-\beta}\right)}^2\left[{\sigma}_1^2+\frac{\sigma_2^2}{r}\right]}{\Delta ^2} $$

$$ {n}_1=\frac{{\left(1.96+0.84\right)}^2\left[{(0.21)}^2+\frac{(0.089)^2}{1}\right]}{(0.2)^2} $$

$$ {n}_1=11 $$

### Statistical analyses

We collected and analysed all endpoint data in accordance with the intention-to-treat principle. Baseline characteristics were presented as numbers and percentages for categorical data, means with standard deviations for normally distributed continuous data, and medians with interquartile ranges for non-normal distributed continuous data. Moreover, Pearson’s chi-squared test and Fisher’s exact test were employed to compare categorical data between the two groups. On the other hand, student’s t-test was used to compare continuous data obtained, with the normal distribution and quantile regression being used to compare continuous data with non-normal distribution between the aforementioned groups. Subsequently, a linear mixed-effects model (LMM) was adopted to evaluate the mean changes in the outcomes, in addition to the adjusted analysis for certain parameters that could affect the outcome. The mean differences were reported to be 95% confidence intervals (CIs) and *P*-values. P-values < 0.05 were considered to be statistically significant. Furthermore, we used a LMM for conducting as-treated analysis in cases of patients whose target dry weight was achieved to confirm the treatment effect. All analysis was performed using Stata software, version 14.2.

## Results

### Enrolment

**Fig. 2:**
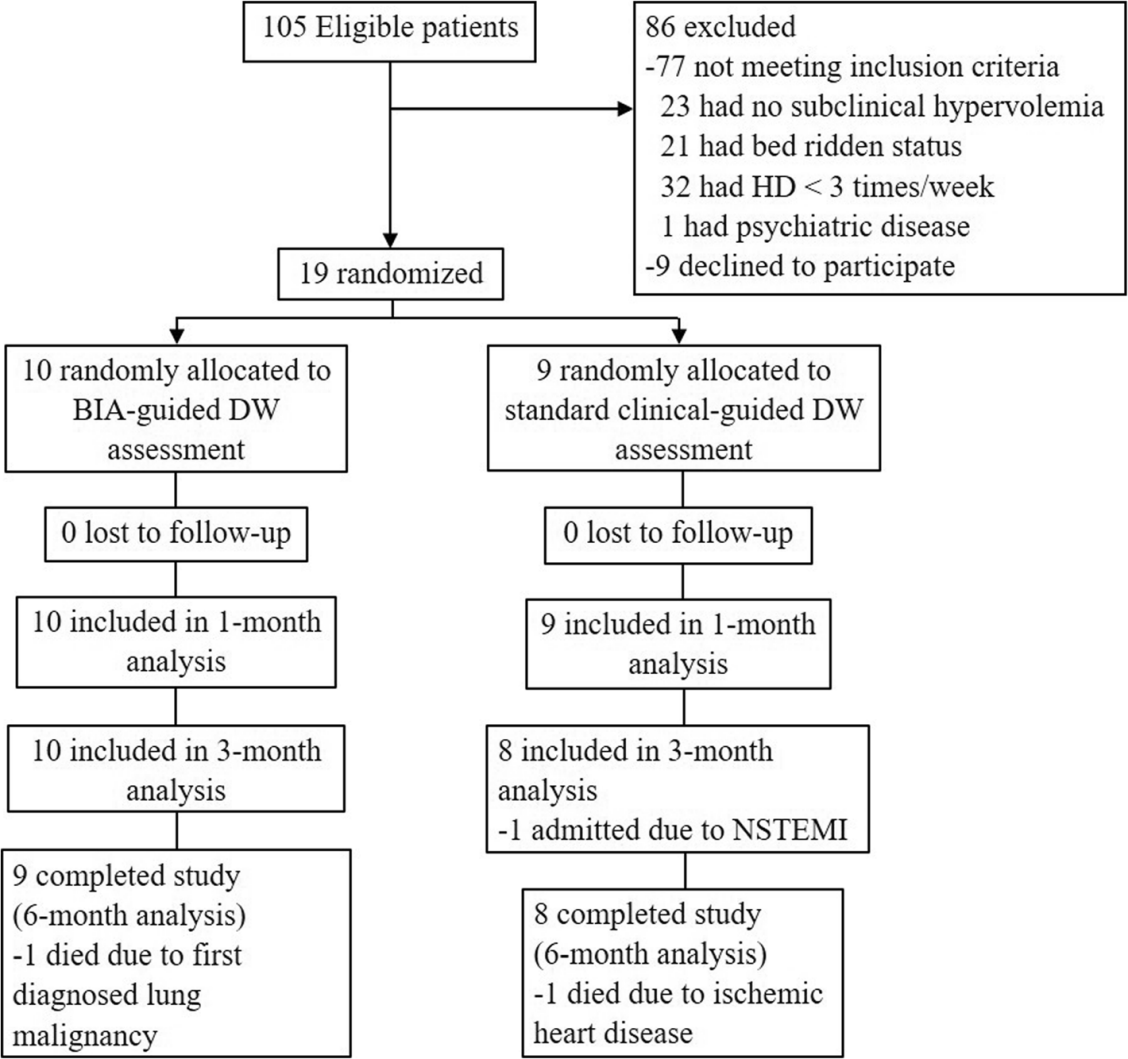
Consolidated Standards of Reporting Trials (CONSORT) flow diagram. Abbreviations: HD, haemodialysis; BIA, bioelectrical impedance analysis; DW, dry weight; NSTEMI, non ST-segment elevation myocardial infarction

For this study, the eligible participants were recruited from July to September 2016. Moreover, a total of 105 patients were screened, and 19 patients were enrolled, wherein 10 patients were randomly assigned to the BIA group and 9 were assigned to the clinical group. The Consolidated Standards of Reporting Trials (CONSORT) flow diagram for this study has been presented in Fig. 2. To elaborate, 17 (89.5%) patients completed the trial at 6 months: 9 (90%) in the BIA group and 8 (88.9%) in the clinical group. The analysis was intention-to-treat and involved all the patients who were randomly assigned.

### Baseline characteristics

The baseline characteristics were similar in the BIA group as well as the clinical one. For the entire study sample, the mean age was 63.53 ± 11.12 years; 42.11% of the participants were male, the mean BMI was 23.45 ± 5.81 kg/m^2^, and equilibrated Kt/V was 2.03 ± 0.43. Nearly 90% of the patients suffered from hypertension, and 58% had diabetes. The majority of the patients were undergoing HD, whereas a minority of them were undergoing online haemodiafiltration. Benzodiazepines were prescribed in 10.53% of cases. Moreover, baseline sleep characteristics obtained through actigraphy (sleep efficiency, sleep duration, WASO), and the sleep questionnaire (PSQI, ESS) scores were similar between the two groups. The mean excess fluid was 0.5 L, with greater fluid retention observed in the BIA group. However, this difference was not statistically significant (Table 1).

**Table 1:** Baseline characteristics of patients at study initiation

	Total (*n* = 19)	BIA group (*n* = 10)	Clinical group (*n* = 9)	*P*-value
Male sex, n (%)	8 (42.11)	5 (50)	3 (33.33)	0.65
Age, yrs	63.53 (11.12)	63.4 (11.69)	63.66 (11.16)	0.90
Comorbidity, n (%)				0.63
-DM	11 (57.89)	6 (60)	5 (55.56)	
-HT	17 (89.47)	9 (90)	8 (88.89)	
-CVD	6 (31.58)	4 (40)	2 (22.22)	
Cause of ESRD, n (%)				0.59
-DN/DKD	9 (47.37)	6 (60)	3 (33.33)	
-HT	5 (26.32)	2 (20)	3 (33.33)	
-Others	5 (26.32)	2 (20)	3 (33.33)	
Benzodiazepines, n (%)	2 (10.53)	0 (0)	2 (22.22)	0.21
Dialysis vintage, yrs	4 (2, 8.5)	3.5 (2, 8)	8 [2, 9]	0.12
RRF, ml	0 (0, 150)	100 (0, 200)	0 (0, 10)	0.52
HD mode, n (%)	16 (84.21)	9 (90)	7 (77.78)	0.58
HD shift, n (%)				0.43
-Morning	6 (31.58)	3 (30)	3 (33.33)	
-Noon	8 (42.11)	3 (30)	5 (55.56)	
-Evening	5 (26.32)	4 (40)	1 (11.11)	
BMI, kg/m^2^	23.45 (5.81)	25.63 (7.00)	21.03 (2.89)	0.08
Pre-PR, bpm	71.21 (8.73)	69.7 (10.64)	72.89 (6.17)	0.44
Pre-SBP, mmHg	145.89 (24.15)	146.8 (24.93)	144.89 (24.72)	0.87
Pre-DBP, mmHg	75.37 (14.33)	72.5 (11.42)	78.56 (17.14)	0.37
eKt/V	2.03 (0.43)	1.90 (0.49)	2.18 (0.31)	0.15
Hb, g/L	108.4 (12.5)	108.5 (13.6)	108.2 (12.0)	0.90
Ferritin, pmol/L	665.11 (355.03, 1467.63)	1142.71 (315.48, 1591.33)	610.96 (357.50, 665.11)	0.09
TSAT, %	0.25 (0.08)	0.27 (0.10)	0.23 (0.05)	0.24
Calcium, mmol/L	2.22 (0.21)	2.23 (0.23)	2.22 (0.20)	0.90
Phosphorus, mmol/L	1.67 (0.48)	1.69 (0.51)	1.66 (0.46)	0.89
Intact PTH, pmol/L	46.65 (24.35, 62.92)	34.73 (17.55, 58.61)	61.04 (39.01, 74.16)	0.37
Albumin, g/L	33.10 (3.0)	32.6 (2.8)	33.7 (3.3)	0.47
B2M, nmol/L	1849.02 (1620.02, 2158.61)	1836.30 (1628.50, 2205.26)	1849.02 (1611.54, 1976.25)	0.87
PSQI	8.53 (3.15)	7.9 (2.42)	9.22 (3.83)	0.38
ESS	7.32 (5.71)	8.4 (5.87)	6.11 (5.42)	0.40
Sleep efficiency, %	71.96 (15.36)	72.52 (13.45)	71.35 (18.08)	0.87
Sleep duration, min	310.61 (84.29)	305.92 (84.31)	315.83 (89.06)	0.81
WASO, min	55.9 (23.27)	57.15 (27.82)	54.51 (18.52)	0.81
Excess fluid assessed by BIA (L)	0.5 (0.11, 1.6)	0.97 (0.35, 1.8)	0.11 (0.09, 1.22)	0.06
ECW/TBW	0.406 (0.005)	0.408 (0.006)	0.404 (0.003)	0.06

Values expressed as the mean (SD) or median (25th, 75th percentiles)

### Study treatment

The median of the additional ultrafiltration fluid in the BIA group was 0.6 L (0.4, 1) in comparison to 0 L (− 0.5, 0) in the clinical group (*P* = 0.19) during the said study period. Furthermore, 60% of the patients in the BIA group achieved the target dry weight. The mean dry weight of the patients assigned to the BIA group was 64.59 kg at the baseline and significantly declined to the lowest point of 63.88 kg at 3 months, whereas no significant changes in dry weight were observed in the clinical group. At the end of our study, 60% of the patients in the BIA group and 44% in the clinical group had a ECW/TBW ratio of less than 0.4, although they were not significantly different (*P* = 0.66).

### Study endpoint

**Fig. 3:**
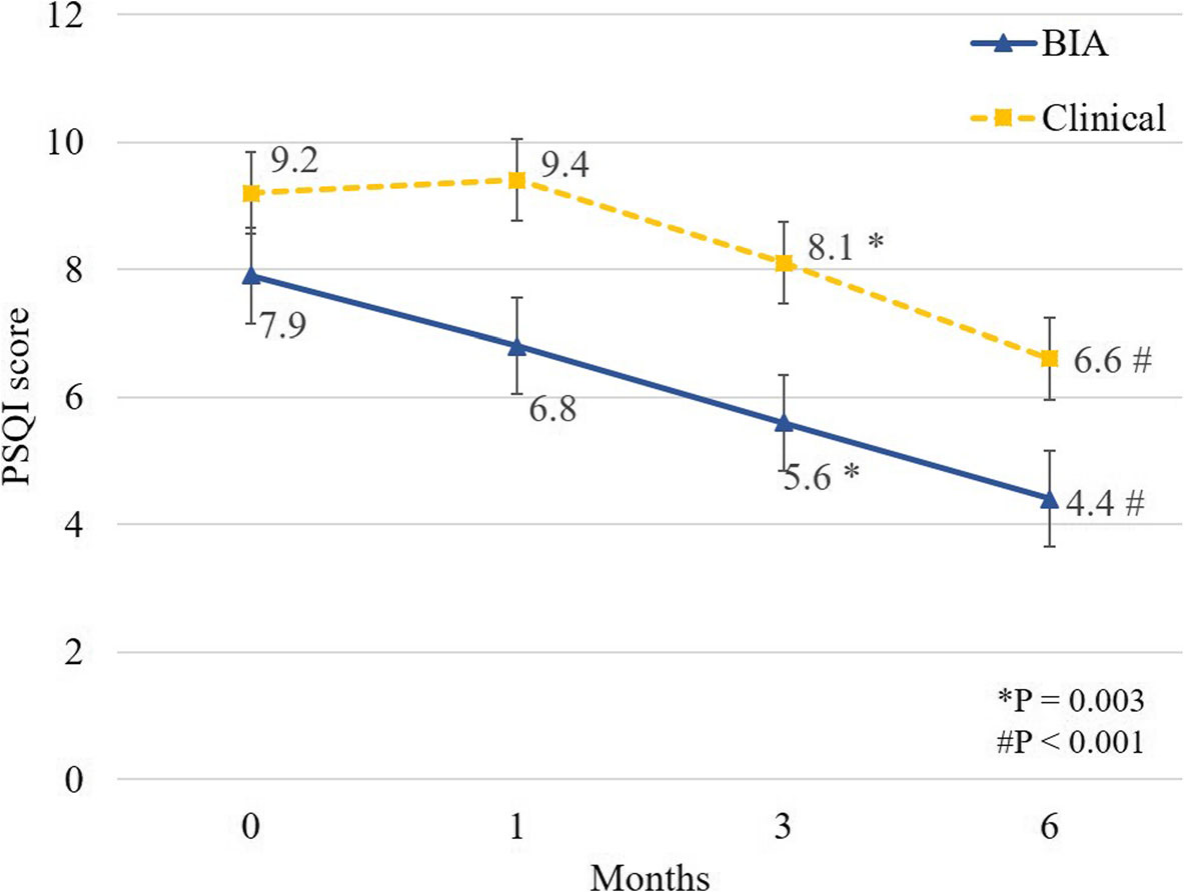
PSQI scores between the BIA and the clinical groups during this study. *P = 0.003. #*P* < 0.001

Changes noted in sleep efficiency, sleep duration, and WASO through actigraphy were not observed to be significantly different between the two groups throughout this study (Table 2 and Additional file 1). The mean changes in the PSQI scores from the baseline to the first month were non-significant between the two groups. However, the BIA group demonstrated a greater decline in terms of their PSQI scores over 3 and 6 months compared to the clinical group, as shown in Fig. 3 (3 months: mean difference − 1.82 [− 3.13 to − 0.51], *P* = 0.006; 6 months: mean difference − 3.16 [− 4.49 to − 1.83], *P* <  0.001). The ESS scores of both the groups also declined at the end of this study. However, the mean change observed between the two groups was non-significant (Table 2). Furthermore, we performed adjustment analysis for factors that could affect the study outcome despite the marginal difference between the said groups, including the baseline PSQI, dialysis vintage, serum ferritin, eKt/V, BMI, and baseline excess fluid (Table 3). The mean changes noted from the baseline of the PSQI score at 3 and 6 months in the BIA group showed significantly greater decline than those observed in case of the clinical group (3 months: mean difference − 1.87 [− 3.11 to − 0.62], *P* = 0.003; 6 months: mean difference − 3.15 [− 4.42 to − 1.88], *P* <  0.001) (Table 3). As observed previously, the mean changes of all the sleep parameters measured through actigraphy and the ESS score were not significantly different between the groups.

**Table 2:** Mean changes in the objective sleep quality (primary outcome) and the subjective sleep quality (secondary outcome) observed during this study

	Baseline	1 month	3 month	6 month
Sleep efficiency				
-BIA group	72.52 (13.45)	71.98 (9.49)	72.88 (13.77)	71.44 (14.79)
-Clinical group	71.35 (18.08)	67.62 (19.09)	66.08 (25.18)	71.60 (24.10)
-Between-group difference (95% CI)	–	−2.05 (−6.05 to 1.96)	−2.21 (−6.29 to 1.87)	−1.37 (−5.53 to 2.79)
-*P*-value	–	0.32	0.29	0.52
Sleep duration				
-BIA group	305.92 (84.31)	288.86 (64.69)	306.75 (83.54)	297.45 (103.85)
-Clinical group	315.83 (89.06)	309.48 (84.13)	299.04 (114.92)	325.19 (125.59)
-Between-group difference (95% CI)				
	–	−11.98 (−42.47 to 18.50)	−6.84 (−37.91 to 24.24)	2.12 (−29.55 to 33.79)
-*P*-value	–	0.44	0.67	0.90
WASO				
-BIA group	57.15 (27.82)	50.15 (18.64)	47.31 (29.39)	46.57 (21.31)
-Clinical group	54.51 (18.52)	62.83 (32.72)	44.98 (25.25)	45.14 (19.95)
-Between-group difference (95% CI)	–	0.26 (−10.06 to 10.58)	−8.45 (−18.97 to 2.07)	−6.67 (−17.38 to 4.04)
-*P*-value	–	0.90	0.12	0.22
PSQI scores				
-BIA group	7.9 (2.42)	6.8 (2.78)	5.6 (2.5)	4.44 (0.88)
-Clinical group	9.22 (3.83)	9.44 (3.13)	8.13 (2.1)	6.62 (3.66)
-Between-group difference (95% CI)	–	−0.47 (−1.76 to 0.81)	−1.82 (−3.13 to − 0.51)	−3.16 (−4.49 to − 1.83)
-*P*-value	–	0.47	0.006	< 0.001
ESS				
-BIA group	8.4 (6.57)	8.8 (6.75)	7.5 (7.56)	7.89 (7.49)
-Clinical group	6.11 (4.65)	6.78 (3.23)	4.88 (3.40)	5.38 (4.93)
-Between-group difference (95% CI)	–	0.53 (−1.10 to 2.15)	−1.12 (−2.77 to 0.54)	−0.82 (−2.51 to 0.86)
-*P*-value	–	0.53	0.19	0.34

PSQI score, ESS score, sleep efficiency, sleep duration, and WASO were expressed as the mean (SD). Changes in the PSQI score, ESS score, sleep efficiency, sleep duration, and WASO from the baseline to 1 month, 3 months, and 6 months were shown with 95% CIs

**Table 3:** Adjusted PSQI scores with clinically relevant variables in various models

	Baseline	1 month	3 month	6 month
PSQI scores				
-BIA group	7.9 (2.42)	6.8 (2.78)	5.6 (2.5)	4.44 (0.88)
-Clinical group	9.22 (3.83)	9.44 (3.13)	8.13 (2.1)	6.62 (3.66)
-Between-group difference (95% CI)	–	−0.47 (−1.76 to 0.81)	−1.82 (−3.13 to − 0.51)	−3.16 (−4.49 to − 1.83)
-*P*-value	–	0.47	0.006	< 0.001
PSQI scores^a^				
-Between-group difference (95% CI)	–	−0.47 (−1.76 to 0.81)	−1.81 (−3.11 to 0.50)	−3.10 (−4.42 to − 1.77)
-*P*-value	–	0.47	0.007	< 0.001
PSQI scores^b^				
-Between-group difference (95% CI)	–	−0.47 (−1.73 to 0.78)	−1.81 (−3.08 to − 0.53)	− 3.11 (−4.41 to − 1.81)
-*P*-value	–	0.46	0.005	< 0.001
PSQI scores^c^				
-Between-group difference (95% CI)	–	−0.47 (− 1.76 to 0.81)	− 1.80 (− 3.10 to − 0.50)	− 3.09 (− 4.42 to − 1.77)
-*P*-value	–	0.47	0.007	< 0.001
PSQI scores^d^				
-Between-group difference (95% CI)	–	− 0.47 (− 1.70 to 0.76)	− 1.87 (− 3.11 to − 0.62)	−3.15 (− 4.42 to − 1.88)
-*P*-value	–	0.45	0.003	< 0.001

^a^ Adjustment for the baseline PSQI, dialysis vintage, serum ferritin, and eKt/V

^b^ Adjustment for the baseline PSQI, dialysis vintage, serum ferritin, eKt/V, and BMI

^c^ Adjustment for the baseline PSQI, dialysis vintage, serum ferritin, eKt/V, and baseline excess fluid

^d^ Adjustment for the baseline PSQI, dialysis vintage, serum ferritin, eKt/V, BMI, and baseline excess fluid

Additionally, we performed as-treated analysis among the participants who achieved the target dry weight in the BIA group in comparison to the clinical group. Moreover, 6 patients were left in the BIA group compared to 9 in the clinical group. In the BIA group, a significant improvement in the PSQI score over 3 and 6 months was demonstrated (3 months: mean difference − 1.75 [− 3.33 to − 0.17], *P* = 0.03; 6 months: mean difference − 3.13 [− 4.74 to − 1.51], *P* <  0.001).

### Adverse events

No serious adverse events occurred during this study. The number of patients who developed intradialytic hypotension (IDH), cramps, and dizziness was not significantly different between the two groups (Table 4).

**Table 4:** Adverse events

	BIA group *n* (%)	Clinical group *n* (%)	*P*-value
Intradialytic hypotension	5 (50)	6 (66.7)	0.65
Cramps	4 (40)	2 (22.2)	0.63
Dizziness	2 (20)	0	0.47

## Discussion

To the best of our knowledge, this is the first randomised controlled trial conducted to provide evidence for bringing about an improvement in the sleep quality of HD patients, using a BIA-guided method. The current results indicate that optimisation of the fluid status by employing BIA significantly improves the subjective sleep quality of chronic HD patients with subclinical hypervolemia.

Patients with advanced kidney diseases, particularly those undergoing maintenance HD, have poor health-related quality of life when compared to that of the general population. Sleep disorders, including OSA, insomnia, and other such sleep disturbances are considered to be important contributors to poor quality of life [5] and increased mortality [7]. Several conditions are likely to play a role in sleep disorders, including iron deficiency anaemia [17], hypercalcemia [18, 19], and systemic inflammation [20, 21]. However, the current evidence in this regard is inconclusive. Hypervolemia is one of the most widely accepted mechanisms responsible for the poor sleep quality of HD patients. Volume overload is a common complication, despite close monitoring by physicians. HD patients with a clinically euvolemic status often exhibit subclinical hypervolemia when assessed through BIA [22]. Overnight redistribution of fluids from the lower extremities to the chest and neck in the supine position could also affect the sleep quality of HD patients [8, 23–25]. Hanly and his colleagues demonstrated an improvement concerning sleep apnoea with nocturnal HD, but it remains unclear whether this effect is caused by the intensification of volume control or the improvement of uremic toxins clearance [9]. Another study ascertained the benefits of reducing fluid overload by conducting HD on OSA severity in patients with ESRD [26]. A lower degree of fluid overload post-HD was significantly correlated to a lower obstructive apnoea-hypopnea index. In contrast to OSA, only one previous study has examined the relationship between volume overload and sleep quality of the HD population. Abreo and his colleagues [10] reported a negative association between the predialysis volume status measured through BIA and the sleep duration of HD patients. However, a causal relationship was not confirmed, since no intervention was performed.

A disparity pertaining to prioritised HD outcomes between health professionals and patients was recently reported, which emphasises the importance of patient-centred outcomes [27]. Sleep quality is one of the core outcomes that can be assessed by both subjective standard questionnaires and objective devices. In our study, the mean changes derived from baseline of sleep parameters that were obtained through actigraphy recordings, which signified the primary outcome, did not show significant differences between the said groups. This could have occurred because the number of targets in both groups was not attained, owing to the strict inclusion criteria, which led to a lack of statistical power. However, the PSQI scores of the BIA group demonstrated a significant amelioration in comparison to those in the clinical group. Since the mean difference in the changes regarding the PSQI scores between the groups was the secondary endpoint, this positive outcome could be theoretically interpreted as random findings. Interestingly, the mean changes in the PSQI scores over 3 and 6 months were still significantly different between the groups, even after adjustment for clinically relevant factors that could possibly affect the outcome, including BMI and baseline excess fluid (which showed unequal values at randomisation, though not statistically significant). Higher BMI and excess fluid at the baseline could probably affect the sleep quality of the participants assigned to the BIA group. In addition, greater fluid volume from the beginning in the BIA group could lead to the presence of more removable fluid through dialysis, which finally resulted in substantial improvement of the PSQI scores for this group. Nevertheless, the adjusted changes on the PSQI scores with these two pertinent variables still showed significantly greater reduction than those in the clinical group. From the aforementioned results, we assumed that optimising the fluid status using BIA somehow significantly improved the subjective sleep quality of chronic HD patients with subclinical hypervolemia. In the current study, the PSQI and ESS were employed to subjectively evaluate the patients’ sleep quality. However, only PSQI scores exhibited a significant improvement. Significantly, the PSQI assesses 6 more domains of sleep (sleep quality, sleep latency, sleep duration, habitual sleep efficiency, sleep disturbances, and use of sleep medication) other than daytime dysfunction. In contrast, the ESS measures only the severity of daytime sleepiness. The current results support the hypothesis that HD patients suffering from subclinical hypervolemia frequently experience fluid redistribution from the legs rostrally while recumbent, which results in oedematous upper airway and disrupted sleep quality. However, the volume of fluid retention might not be sufficient to cause OSA and daytime sleepiness. Another potential explanation in this regard is that ESS scores could be confounded by stress during HD in the daytime.

In addition to the small number of participants in this study, another reason the sleep parameters assessed through actigraphy recordings are not significantly different between the two groups is that objectively and subjectively measured sleep are only moderately correlated [28, 29], and changes in PSQI scores, although statistically significant, were relatively minor.

Subjective sleep quality is reported to be a predictor of outcomes, including survival, in HD patients [30]. Our study showed that the reduction of subclinical fluid overload plays a major role, as the BIA group exhibited a rapid and constant improvement in their subjective sleep quality over the study period (Fig. 3), although dialysis adequacy was comparable in this regard. However, our study was potentially limited in several ways. First, this study did not include an objective OSA assessment. Although we screened participants for OSA symptoms using the Berlin questionnaire [31], this measure has been noted to have poor sensitivity and specificity in terms of detecting OSA in HD patients, when compared to objective OSA assessment [32]. However, the average BMI of the participants was 23, and 26% of them were at the risk of OSA according to the said questionnaire. Second, the follow-up period might have been too short, although we did observe significant improvement in the PSQI scores. Extending this study for a longer period might reveal the benefits of the said intervention more clearly on the subjective as well as objective outcomes. Third, a relatively small sample size, due to markedly strict inclusion criteria, may result in less statistical power to detect objective sleep quality improvement. Last but not least, this study included haemodialysis patients with subclinical hypervolemia defined by BIA, which can detect even minimal fluid excess sometimes less than 0.5 L; consequently, findings from this study may not be applicable to general haemodialysis patients.

Employing BIA as a tool to guide ultrafiltration and dry weight adjustment in case of HD patients has been validated previously [33–35]. The current study provides new evidence to further expand current understandings concerning the pathophysiology of poor sleep quality in patients with maintenance HD, which suggests that better hydration status management using BIA can improve the subjective sleep quality in subclinical hypervolemic HD patients. However, extrapolating the aforementioned observations to other populations, not included in the study, should be used with caution.

## Conclusions

Optimisation of the fluid status using BIA did not significantly improves sleep actigraphy; however, it significantly ameliorates the subjective sleep quality in subclinical hypervolemic chronic HD patients. However, clinical trials with larger study samples and longer research durations, in addition to studies designed to use mean changes in PSQI scores as the primary outcome, will be needed to confirm these promising results in the future.

## Additional file

Additional file 1: BEDTIME study LMM analysis. (DOC 40 kb)

## Data Availability

All data generated or analysed during this study have been included in this published article and Additional file 1.

## Abbreviations

B2M: Beta-2 microglobulin
BIA: Bioelectrical impedance analysis
BMI: Body mass index
BP: Blood pressure
CVD: Cardiovascular disease
DKD: Diabetic kidney disease
DM: Diabetes mellitus
DN: Diabetic nephropathy
ECW: Extracellular water
eKt/V: Equilibrated Kt/V
ESRD: End-stage renal disease
ESS: Epworth Sleepiness Scale
Hb: Haemoglobin
HD: Haemodialysis
HT: Hypertension
IDH: Intradialytic hypotension
OSA: Obstructive sleep apnoea
Pre-DBP: Predialysis diastolic blood pressure
Pre-PR: Predialysis pulse rate
Pre-SBP: Predialysis systolic blood pressure
PSG: Polysomnography
PSQI: Pittsburgh Sleep Quality Index
PTH: Parathyroid hormone
RRF: Residual renal function
TBW: Total body water
TSAT: Transferrin saturation
UF: Ultrafiltration
WASO: Wake after sleep onset
