# Supplementary material for: Effect of bioelectrical impedance analysis-guided dry weight adjustment, in comparison to standard clinical-guided, on the sleep quality of chronic haemodialysis patients (BEDTIME study): a randomised controlled trial
Source: BMC Nephrol. 2019 Sep 2;20:211. doi: 10.1186/s12882-019-1405-z (PMC6717999; doi:10.1186/s12882-019-1405-z)
Supplement: Supplementary file 1 — BEDTIME study LMM analysis. (DOC 40 kb) [file 12882_2019_1405_MOESM1_ESM.doc]

. *PSQI

Mixed-effects ML regression Number of obs = 73

Group variable: id Number of groups = 19

Obs per group:

min = 2

avg = 3.8

max = 4

Wald chi2(10) = 113.82

Log likelihood = -151.67012 Prob > chi2 = 0.0000

--------------------------------------------------------------------------------

psqi | Coef. Std. Err. z P>|z| [95% Conf. Interval]

---------------+----------------------------------------------------------------

time |

1 | -.4736842 .6269355 -0.76 0.450 -1.702455 .7550869

3 | -1.86636 .6366746 -2.93 0.003 -3.11422 -.6185008

6 | -3.149638 .6465608 -4.87 0.000 -4.416874 -1.882402

|

1.group | -1.294825 .7224287 -1.79 0.073 -2.710759 .1211095

psqi_base | .5270935 .0848397 6.21 0.000 .3608106 .6933763

vintageyr_base | .2294924 .0786019 2.92 0.004 .0754355 .3835493

ferritin0 | -.0006413 .0007535 -0.85 0.395 -.0021182 .0008356

ektv | -.2213343 .9160132 -0.24 0.809 -2.016687 1.574019

bmi | .2367172 .0886089 2.67 0.008 .063047 .4103874

exfl | -.8968814 .4849082 -1.85 0.064 -1.847284 .0535212

_cons | -.582269 3.605492 -0.16 0.872 -7.648903 6.484365

--------------------------------------------------------------------------------

------------------------------------------------------------------------------

Random-effects Parameters | Estimate Std. Err. [95% Conf. Interval]

-----------------------------+------------------------------------------------

id: Identity |

var(_cons) | 4.14e-23 4.75e-22 6.97e-33 2.46e-13

-----------------------------+------------------------------------------------

var(Residual) | 3.733958 .6180539 2.699457 5.164905

------------------------------------------------------------------------------

LR test vs. linear model: chibar2(01) = 0.00 Prob >= chibar2 = 1.0000

. ******************************

. *epworth

Mixed-effects ML regression Number of obs = 73

Group variable: id Number of groups = 19

Obs per group:

min = 2

avg = 3.8

max = 4

Wald chi2(10) = 16.09

Log likelihood = -193.67359 Prob > chi2 = 0.0970

--------------------------------------------------------------------------------

epworth | Coef. Std. Err. z P>|z| [95% Conf. Interval]

---------------+----------------------------------------------------------------

time |

1 | .5263158 .8283311 0.64 0.525 -1.097183 2.149815

3 | -1.089683 .8447914 -1.29 0.197 -2.745444 .5660781

6 | -.8219788 .8609738 -0.95 0.340 -2.509456 .8654988

|

1.group | -.1147307 2.93649 -0.04 0.969 -5.870145 5.640684

psqi_base | .0574955 .3551824 0.16 0.871 -.6386492 .7536401

vintageyr_base | .0185768 .3296472 0.06 0.955 -.6275198 .6646735

ferritin0 | .0047594 .0030975 1.54 0.124 -.0013115 .0108304

ektv | -.35721 3.819734 -0.09 0.925 -7.843752 7.129331

bmi | -.0870314 .3587029 -0.24 0.808 -.7900761 .6160133

exfl | 2.558485 1.910331 1.34 0.180 -1.185694 6.302664

_cons | 4.97151 14.72221 0.34 0.736 -23.8835 33.82652

--------------------------------------------------------------------------------

------------------------------------------------------------------------------

Random-effects Parameters | Estimate Std. Err. [95% Conf. Interval]

-----------------------------+------------------------------------------------

id: Identity |

var(_cons) | 15.04986 5.441815 7.408842 30.57137

-----------------------------+------------------------------------------------

var(Residual) | 6.518258 1.253344 4.471577 9.501722

------------------------------------------------------------------------------

LR test vs. linear model: chibar2(01) = 44.56 Prob >= chibar2 = 0.0000

. ******************************

. *slpeff

Mixed-effects ML regression Number of obs = 73

Group variable: id Number of groups = 19

Obs per group:

min = 2

avg = 3.8

max = 4

Wald chi2(10) = 15.40

Log likelihood = -262.55064 Prob > chi2 = 0.1183

--------------------------------------------------------------------------------

slpeff | Coef. Std. Err. z P>|z| [95% Conf. Interval]

---------------+----------------------------------------------------------------

time |

1 | -2.048177 2.043721 -1.00 0.316 -6.053797 1.957442

3 | -2.12509 2.084727 -1.02 0.308 -6.21108 1.960899

6 | -1.260897 2.124945 -0.59 0.553 -5.425712 2.903918

|

1.group | -1.355352 8.456707 -0.16 0.873 -17.93019 15.21949

psqi_base | -2.198593 1.024028 -2.15 0.032 -4.205652 -.191535

vintageyr_base | -1.718212 .9504678 -1.81 0.071 -3.581095 .1446705

ferritin0 | .0060432 .0089243 0.68 0.498 -.011448 .0235345

ektv | 11.2526 11.01104 1.02 0.307 -10.32865 32.83384

bmi | -1.011093 1.032477 -0.98 0.327 -3.034712 1.012525

exfl | 6.273958 5.491468 1.14 0.253 -4.489121 17.03704

_cons | 93.55784 42.4009 2.21 0.027 10.45361 176.6621

--------------------------------------------------------------------------------

------------------------------------------------------------------------------

Random-effects Parameters | Estimate Std. Err. [95% Conf. Interval]

-----------------------------+------------------------------------------------

id: Identity |

var(_cons) | 128.8223 45.29131 64.6734 256.5998

-----------------------------+------------------------------------------------

var(Residual) | 39.67955 7.63731 27.21022 57.86308

------------------------------------------------------------------------------

LR test vs. linear model: chibar2(01) = 55.75 Prob >= chibar2 = 0.0000

. ******************************

. **actslp

Mixed-effects ML regression Number of obs = 73

Group variable: id Number of groups = 19

Obs per group:

min = 2

avg = 3.8

max = 4

Wald chi2(10) = 13.88

Log likelihood = -403.09246 Prob > chi2 = 0.1785

--------------------------------------------------------------------------------

actslp | Coef. Std. Err. z P>|z| [95% Conf. Interval]

---------------+----------------------------------------------------------------

time |

1 | -11.98462 15.54849 -0.77 0.441 -42.45911 18.48987

3 | -6.23375 15.85071 -0.39 0.694 -37.30056 24.83306

6 | 2.797879 16.14936 0.17 0.862 -28.85428 34.45004

|

1.group | -5.505994 43.24001 -0.13 0.899 -90.25486 79.24288

psqi_base | -4.49776 5.216581 -0.86 0.389 -14.72207 5.726551

vintageyr_base | -8.297838 4.840835 -1.71 0.087 -17.7857 1.190023

ferritin0 | -.0084903 .0455648 -0.19 0.852 -.0977956 .080815

ektv | 96.34558 56.12004 1.72 0.086 -13.64767 206.3388

bmi | -3.251439 5.287661 -0.61 0.539 -13.61506 7.112185

exfl | 25.09185 28.24225 0.89 0.374 -30.26195 80.44564

_cons | 261.2064 216.7441 1.21 0.228 -163.6042 686.017

--------------------------------------------------------------------------------

------------------------------------------------------------------------------

Random-effects Parameters | Estimate Std. Err. [95% Conf. Interval]

-----------------------------+------------------------------------------------

id: Identity |

var(_cons) | 3017.878 1174.159 1407.753 6469.59

-----------------------------+------------------------------------------------

var(Residual) | 2296.679 440.9607 1576.414 3346.034

------------------------------------------------------------------------------

LR test vs. linear model: chibar2(01) = 28.03 Prob >= chibar2 = 0.0000

. ******************************

. *waso

Mixed-effects ML regression Number of obs = 73

Group variable: id Number of groups = 19

Obs per group:

min = 2

avg = 3.8

max = 4

Wald chi2(10) = 7.22

Log likelihood = -322.04692 Prob > chi2 = 0.7043

--------------------------------------------------------------------------------

waso | Coef. Std. Err. z P>|z| [95% Conf. Interval]

---------------+----------------------------------------------------------------

time |

1 | .258302 5.262387 0.05 0.961 -10.05579 10.57239

3 | -8.392148 5.363378 -1.56 0.118 -18.90417 2.119879

6 | -6.606627 5.46346 -1.21 0.227 -17.31481 4.101558

|

1.group | 1.032735 13.21191 0.08 0.938 -24.86213 26.9276

psqi_base | -.3632797 1.591542 -0.23 0.819 -3.482646 2.756086

vintageyr_base | -.0378291 1.476779 -0.03 0.980 -2.932263 2.856604

ferritin0 | -.0138478 .0139142 -1.00 0.320 -.0411191 .0134235

ektv | 15.799 17.12525 0.92 0.356 -17.76587 49.36387

bmi | .5500188 1.61653 0.34 0.734 -2.618322 3.718359

exfl | 3.627161 8.648145 0.42 0.675 -13.32289 20.57721

_cons | 16.96687 66.21698 0.26 0.798 -112.816 146.7498

--------------------------------------------------------------------------------

------------------------------------------------------------------------------

Random-effects Parameters | Estimate Std. Err. [95% Conf. Interval]

-----------------------------+------------------------------------------------

id: Identity |

var(_cons) | 268.2679 112.4804 117.9434 610.1882

-----------------------------+------------------------------------------------

var(Residual) | 263.0808 50.91318 180.0358 384.4319

------------------------------------------------------------------------------

LR test vs. linear model: chibar2(01) = 19.05 Prob >= chibar2 = 0.0000

. ******************************
